# Prevention of deep vein thrombosis in postoperative orthopedic patients: a hybrid meta-analysis and clinical case study

**DOI:** 10.3389/fmed.2025.1603191

**Published:** 2025-08-14

**Authors:** Fenglan Liu, Juan Tan, Yulin Pan

**Affiliations:** ^1^Department of Orthopedics, Sichuan Dazhou Central Hospital, Dazhou, China; ^2^Department of Critical Care Medicine, Sichuan Dazhou Central Hospital, Dazhou, China

**Keywords:** deep vein thrombosis (DVT), orthopedic surgery, D-dimer, nursing interventions, prevention strategies

## Abstract

**Background:**

Deep vein thrombosis (DVT) is a significant complication, which often comes along with orthopedic surgery, and leads risks of severe outcomes such as pulmonary embolism. Effective prevention strategies are urgently expected to promote postoperative recovery and reduce complications.

**Objective:**

This study aims to measure the effectiveness of nursing interventions in preventing postoperative DVT among the patients with orthopedic surgeries, with the focus on outcomes such as DVT incidence, recovery time, and patient satisfaction. This hybrid study integrates a PRISMA-compliant meta-analysis of six independent studies with a prospective clinical case report, bridging population-level evidence and real-world nursing practice.

**Methods:**

Nursing intervention details (early mobilization and patient education) were firstly collected and assessed in postoperative patients in this research. Key metrics (DVT occurrence, D-dimer level, and patient-reported outcomes) were further analyzed to determine the impact of these interventions.

**Results:**

Following the nursing interventions, patients demonstrated a lower DVT incidence, the recovery rates as well as patient satisfaction were enhanced, and the symptoms of lower extremity swelling got reduced, despite challenges in adherence to the protocols.

**Conclusion:**

Nursing interventions play a critical role in DVT prevention in postoperative care for the patients after orthopedic surgeries. These findings support the integration of standardized nursing practices to optimize postoperative outcomes after orthopedic surgeries and highlight the need for addressing adherence barriers to maximize their effectiveness.

## 1 Introduction

Deep vein thrombosis (DVT) is a common serious complication following orthopedic surgeries (1), characterized by the formation of blood clots in the deep veins (2), most often in the lower extremities (3). According to a study by Anderson et al., the DVT incidence could be as high as 40–60% if prophylaxis efforts are not carried after the major orthopedic surgeries (4). If left untreated, DVT can become life-threatening, such as leading pulmonary embolism (PE), which significantly increases morbidity and mortality among the patients after the orthopedic surgeries (5), especially those in the lower limbs like total hip arthroplasty (THA) and knee arthroplasty (KA), are associated with a higher risk of DVT for the reasons like prolonged immobility, surgical trauma, and hypercoagulability induced by the inflammatory response to surgery (6–8). Additionally, patients with comorbid conditions such as diabetes mellitus or obesity are at even greater risk, further underscoring the importance of effective preventive measures (9).

Research on DVT prevention after surgery is warranted because it is crucial not only for improving postoperative outcomes but also for reducing healthcare costs associated with prolonged hospitalization and complication management (10). Although anticoagulants are routinely prescribed to prevent DVT, they also carry the risk of bleeding complications (11, 12). Therefore, non-pharmacological interventions, particularly nursing interventions, have gained increasing attention as essential components of postoperative care (13). These interventions including early mobilization, patient education, and regular monitoring (14, 15), aim to address the modifiable risk factors associated with DVT, such as immobility and lack of patient awareness. According to a study by D’Astous et al. (16), the DVT incidence will become higher if prophylaxis is omitted after major orthopedic surgeries. Even with pharmacological prophylaxis such as heparin, significant residual risk might still remain (17). These findings highlight the critical need for complementary nursing interventions to address modifiable risk factors such as immobility and patient-specific vulnerabilities.

Despite the availability of effective prevention strategies, challenges remain in implementing these measures consistently across healthcare settings. Variability in the intensity and frequency of nursing interventions, as well as poor patient adherence due to pain or limited motivation, can undermine their effectiveness (18, 19). For example, in a clinical case tracked in this study, a 52-year-old male patient with a normal BMI underwent left forearm fracture surgery under general anesthesia. Despite having no history of hypertension or thrombosis, the patient developed DVT one week postoperatively, with ultrasound findings indicating thrombi in the lower extremity deep veins, right atrium, and vena cava, accompanied by a large-area acute pulmonary embolism. This case highlights the severity of DVT in orthopedic postoperative patients and underscores the need for effective preventive measures.

D-dimer has traditionally been used as a thrombosis biomarker (20), though its reliability postoperatively is limited due to surgical interference (21). To bridge evidence and practice, this study combines a meta-analysis with a clinical case (detailed in Results 3.3), which highlights adherence challenges in real-world nursing care.

To reconcile aggregate evidence with clinical reality, this study employs a hybrid design: a systematic meta-analysis of randomized controlled trials (RCTs) and cohort studies (*n* = 6, per PRISMA guidelines) combined with a prospectively tracked 52-year-old patient case from our institution. This dual approach highlights both population-level efficacy of nursing interventions and on-the-ground challenges in high-risk patients.

This study aims to evaluate nursing interventions such as active early mobilization, early ambulation and supervised walking in preventing DVT. Active mobilization strategies, including patient-initiated movements, were prioritized due to their established role in promoting venous return. By conducting a systematic review of RCTs and cohort studies, this research was intended to analyze data from multiple studies to assess the impact of nursing interventions on DVT prevention.

## 2 Materials and methods

### 2.1 Research strategy

This hybrid study adheres to PRISMA standards for the meta-analysis component for the clinical case report, integrating evidence synthesis with real-world observation. A systematic search strategy was designed and documented for this systematic review of nursing interventions in orthopedic postoperative patients. We strictly adhered to the PRISMA guidelines to ensure quality (22), and the study protocol was not registered in a public registry.

**Figure 1:**
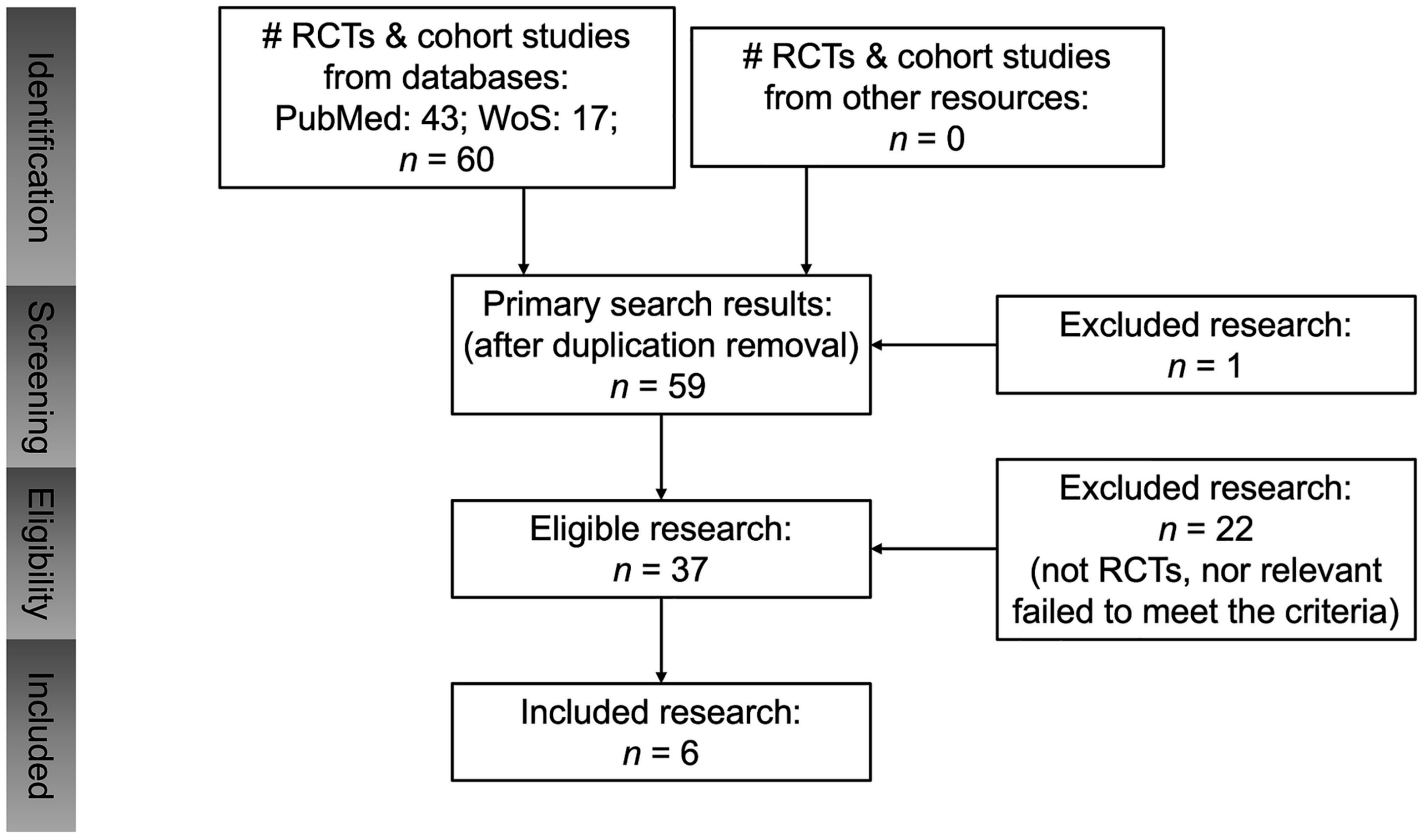
Flowchart of study.

As shown in Figure 1, the databases PubMed and WoS (Web of Sciences) were used for their comprehensive coverage of biomedical literature, including RCTs and cohort studies on nursing interventions. The results were searched by adopting the Boolean search strategy: (orthopedic surgery) AND (postoperative nursing) AND (DVT OR deep vein thrombosis) AND (limb function recovery OR rehabilitation). We included English and Chinese literature in the last 10 years, from 2015 to 2025.

After exporting initial results, articles were screened by titles and abstracts for relevance. Then, full texts were examined to collect detailed outcomes from relevant ones. This process ensures robust data for further analysis, aiding in understanding effective nursing strategies.

### 2.2 Inclusion and exclusion criteria

Inclusion criteria: English and Chinese studies (2015–2025) on nursing interventions for orthopedic postoperative patients, with full-text data available, regardless of authorship or institutional location. Title and abstract screening were performed independently by two reviewers (FL and JT) in a double-blinded manner using a shared spreadsheet. Discrepancies were resolved by a third reviewer (YP), ensuring objectivity and reliability. The studies included in this research were selected based on the above inclusion criteria focusing on orthopedic postoperative patients. Inclusion was restricted to RCTs and cohort studies, with the incidence of DVT calculated for each eligible study.

In contrast, only literature that reports both postoperative complications and recovery status was included, while those that fail to do so were excluded. Additionally, only English-language publications providing comprehensive data within the research were considered, and those not meeting these criteria were omitted from this analysis.

### 2.3 Data extraction

Data was extracted from the included studies using a standardized form by two independent reviewers, with discrepancies resolved by a third. Key variables extracted included study details (author, year, sample size), patient demographics (age, gender) (Table 1), to assess patients’ basic health conditions.

**Table 1:** Demographic features of the included patients to be researched.

Reference	Study design	Surgery type	Treatment method	Control group (no care)				Observational group (with care)			
File name				Case number	Age (mean ± SD)	Male: Female	DVT Cases	Case number	Age (mean ± SD)	Male: Female	DVT cases
1. Wang et al. (2016) (20)	RCT	Lower limb surgery	Active ankle movement	78	52.2 ± 6.9	65:13	14	96	53.5 ± 6.2	78:18	7
2. Li et al. (2016) (21)	RCT	Lower limb surgery	Active ankle movements	97	54.3 ± 6.4	72:24	8	96	53.6 ± 5.4	77:19	1
3. Yang et al. (2016) (22)	Cohort Study	Femoral shaft fracture after intramedullary nail fixation surgery	Lower LIMB REHABILITATION TRAINING	80	67 ± 6	51:29	18	80	67 ± 6	45:35	7
4. Liu et al. (2017) (14)	Cohort Study	Lumbar fusion surgery	Lower limb rehabilitation training	300	64 ± 7	131:169	63	240	64 ± 7	116:124	26
5. Li et al. (2019) (15)	Cohort Study	Orthopedic surgery	Lower limb rehabilitation training	53	61.07 ± 13.68	28:25	23	53	60.24 ± 14.09	29:24	9
6. Liu et al. (2021) (23)	RCT	Hip fracture surgery	Lower limb rehabilitation training	68	73.4 ± 7.9	35:33	6	68	75.2 ± 7.1	39:29	0

#### 2.3.1 Study subjects

As shown in Table 1, this study population consisted of postoperative orthopedic patients, with demographic and clinical data sourced from multiple studies. Patients’ age varied across studies. While detailed medical histories were not presented in the table, relevant information potentially influencing outcomes, such as comorbidities and prior health conditions, was analyzed during the study. Patients were stratified into intervention and control groups to compare the impact of different nursing strategies on recovery outcomes.

#### 2.3.2 Intervention measures

Nursing interventions focused on active early mobilization, including: (1) early ambulation: Standing under medical supervision within 24 h post-surgery, with intensity gradually increased; (2) ankle pump exercises; (3) passive mobilization such as assisted joint flexion and extension, was not included in this protocol. Passive mobilization was excluded to isolate the effect of patient-initiated movements, as prior studies show active exercises more effectively enhance maximum venous outflow (MVO) and reduce thrombosis risk.

Nursing interventions also included regular turning care: Patients were repositioned every 2 h to alleviate pressure on bony prominences, reducing the risk of pressure ulcers. This care was independent of mobilization protocols and applied uniformly across groups.

Patient compliance posed a challenge, often hindered by postoperative pain or apprehension. To address this, healthcare staff provided detailed explanations of the interventions’ importance and methods before implementation. Health education initiatives enhanced patient awareness and cooperation. Continuous monitoring of emotional and physical responses allowed for timely support and adjustments, fostering active participation in the recovery process.

For patients undergoing lower limb surgeries such as THA, KA and thighbone surgery, early mobilization interventions included:

(1) Active ankle movements (dorsiflexion, varus, plantar flexion, valgus) with specified angles.
(2) Lower limb rehabilitation training (lying flat exercises, limb massage, ankle pumps, knee-pressing, quadriceps contractions, knee-hip bending) with defined durations and repetitions.

#### 2.3.3 Observation indicators

The incidence of DVT was selected as the key indicator in this research, as a common and serious postoperative complication. We recorded the number of DVT cases in both the control and intervention groups to assess the effectiveness of the nursing interventions. This comparison helped us understand if the interventions reduced the risk of DVT and improved patient outcomes after surgery.

### 2.4 Risk of biases detection

Publication bias can skew analysis results. In this research, a funnel plot was created to spot potential publication biases. It shows the relationship between the effect size (risk difference of DVT incidence) and its precision (standard error). Data from all included studies were used. Symmetric data points around the pooled effect on the plot suggest low publication bias. Besides visual inspection, Egger’s regression test was done for a more objective assessment.

### 2.5 Quality assessment

The Cochrane Risk of Bias tool was used to assess study quality across selection, performance, detection, attrition, and reporting bias domains. In this research, two independent clinical doctors reviewed each study. They evaluated elements like random sequence generation and allocation concealment for selection bias, blinding for performance and detection biases, incomplete data for attrition bias, and selective reporting. Only studies where the two doctors’ opinions fully agreed were included. This ensured study quality. Finally, each study was classified as “low risk”, “high risk”, or “unclear risk” of bias.

### 2.6 Statistical analysis

This research was conducted using specialized software such as Review Manager (RevMan, version 5.4) for in-depth statistical analysis. For the case number of the DVT patients, which is defined as the dichotomous outcome, odds ratio (OR) with 95% CIs was applied.

The *I*^2^ statistic was used to evaluate the heterogeneity among studies. When the *I*^2^ value exceeded 50%, indicating significant heterogeneity, a random effects model was adopted. Additionally, sensitivity analysis was performed to verify the robustness of the results.

## 3 Results

### 3.1 Risk difference in orthopedic care studies

**Figure 2:**
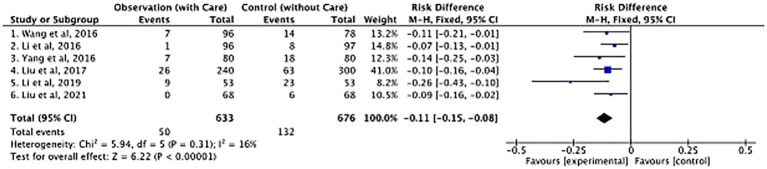
Forest plot of postoperative DVT between the observation group (with care) and the control group (without care) in orthopedic studies.

The risk difference in DVT incidence between the observation group (receiving nursing care) and the control group (without nursing care) was analyzed across multiple studies. As presented in Figure 2, data from six different studies were included. Each study’s results contributed to understanding the impact of nursing interventions on DVT prevention. The overall risk difference among the 6 included publications was −0.11 (95% CI [−0.15, −0.08]) with Z-value of 6.22 (*p* < 0.00001). This significant result strongly suggests that nursing interventions play a crucial role in decreasing the incidence of DVT in postoperative orthopedic patients. The low heterogeneity (Chi^2^ = 5.94, df = 5, *p* = 0.31; I^2^ = 16%) among the studies further validates the consistency of this finding.

### 3.2 Risks of biases detection

**Figure 3:**
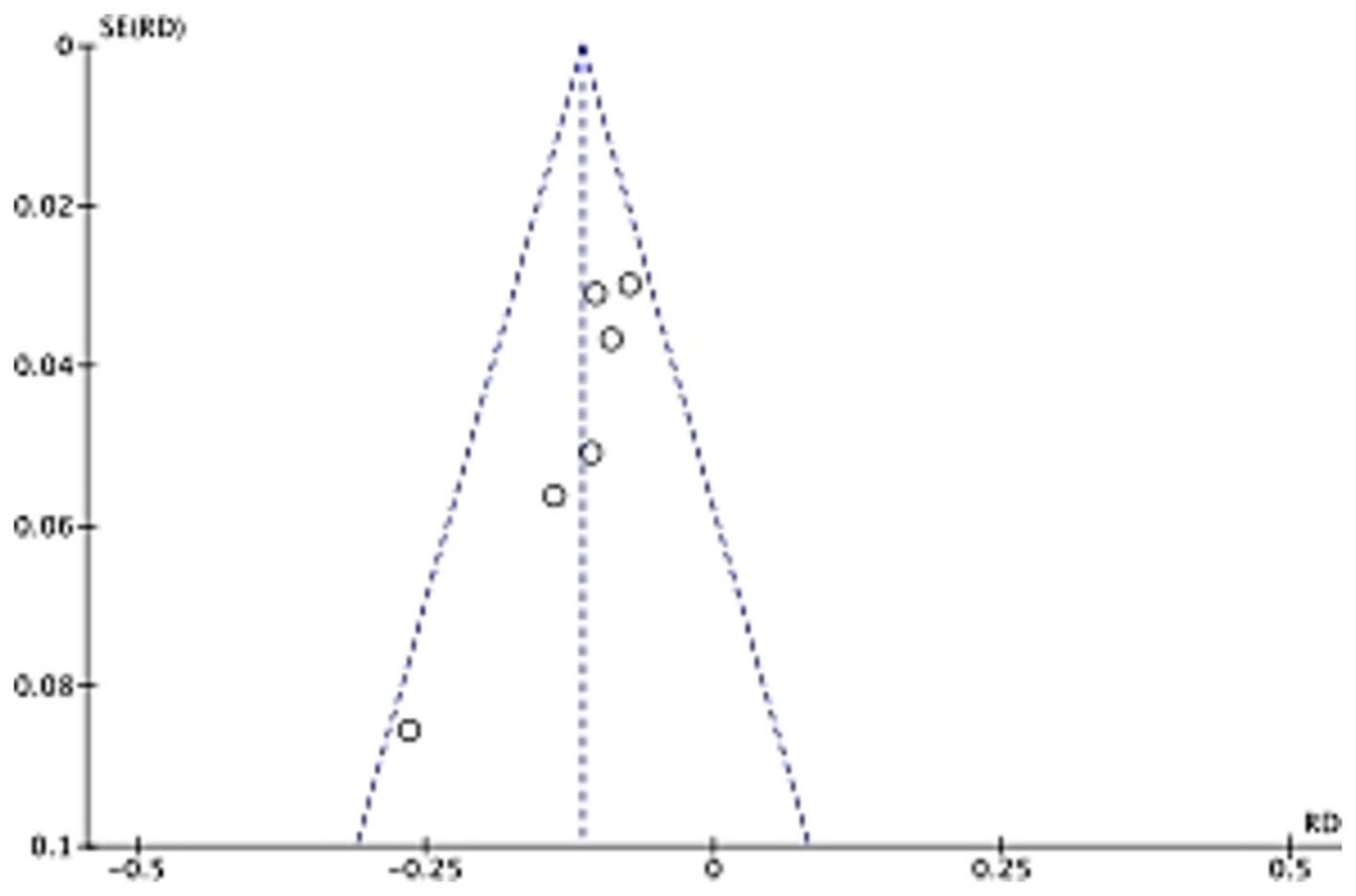
Funnel plot of postoperative DVT between the observation group (with care) and the control group (without care) in orthopedic studies.

To assess potential biases in the included studies, a funnel plot was utilized, as shown in Figure 3. The funnel plot is a graphical representation of the relationship between the effect size (risk difference in this case) and its precision (standard error).

In an ideal situation without publication bias, the studies should be symmetrically distributed around the pooled effect estimate. In our analysis, visual inspection of the funnel plot indicates a relatively symmetric distribution, suggesting a low likelihood of significant publication bias. However, to confirm this, Egger’s regression test was also performed. The results of these analyses are crucial for ensuring the reliability of the overall study findings, as biases can distort the true effect of nursing interventions on DVT prevention.

### 3.3 DVT occurrence and D-dimer dynamics in a postoperative case

To contextualize the meta-analysis findings (−0.11 risk difference, *p* < 0.00001), a 52-year-old male patient with a normal BMI underwent surgery for left forearm fractures under general anesthesia, developed DVT one week postoperatively. Despite no history of hypertension or thrombosis, he developed DVT one week postoperatively. Color Doppler ultrasound revealed thrombi in the lower extremity deep veins, right atrium, and inferior vena cava, accompanied by a large-area acute pulmonary embolism. On February 25, 2024, ultrasound imaging detected flocculent echoes in the right atrium and inferior vena cava, along with right heart enlargement and mild tricuspid regurgitation. By February 27, persistent right heart dilation and aortic sinus widening were observed, confirming the severity of thrombotic complications.

**Figure 4:**
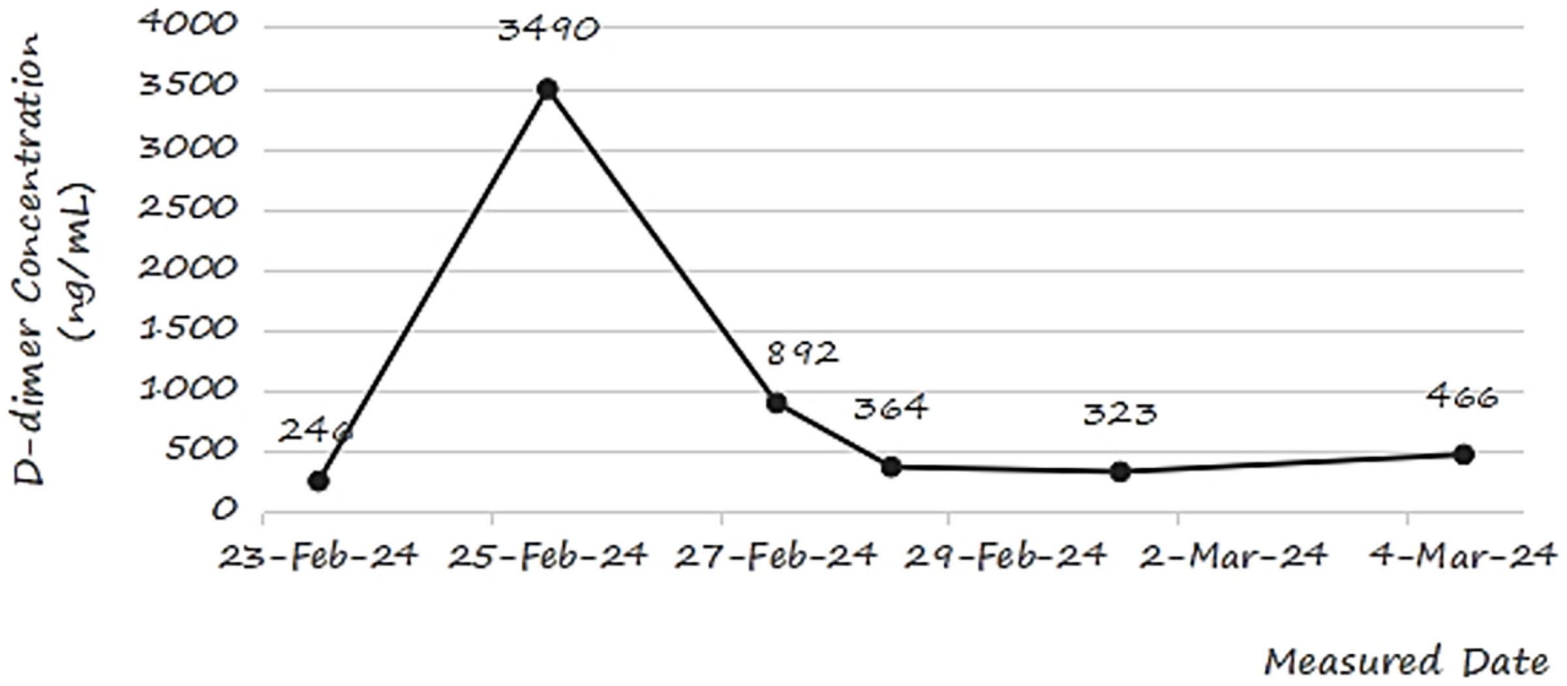
Changes in D-dimer concentration of a postoperative orthopedic patient during the recovery period.

Concurrently, dynamic tracking of D-dimer levels (Figure 4) revealed significant fluctuations. Levels surged from 246 ng/mL on February 23 to a peak of 3,490 ng/mL on February 25, coinciding with the clinical diagnosis of DVT. This sharp rise reflects active fibrin degradation during thrombus formation, consistent with D-dimer’s role as a biomarker for hypercoagulability and VTE (20). Subsequent declines to 892 ng/mL (February 27), 364 ng/mL (February 28), and 323 ng/mL (March 1) suggested partial thrombus resolution or transient stabilization of coagulation. However, a rebound to 466 ng/mL on March 4 highlighted the persistent risk of thrombotic recurrence, necessitating vigilant monitoring.

This case underscores the interplay between clinical manifestations and laboratory biomarkers in postoperative DVT management. Despite nursing interventions aimed at early mobilization, the patient’s poor adherence due to postoperative pain likely contributed to delayed recovery and coagulation abnormalities. These findings emphasize the need for tailored strategies to enhance patient compliance and integrate biomarker monitoring into routine postoperative care.

### 3.4 Quality assessment results

**Figure 5:**
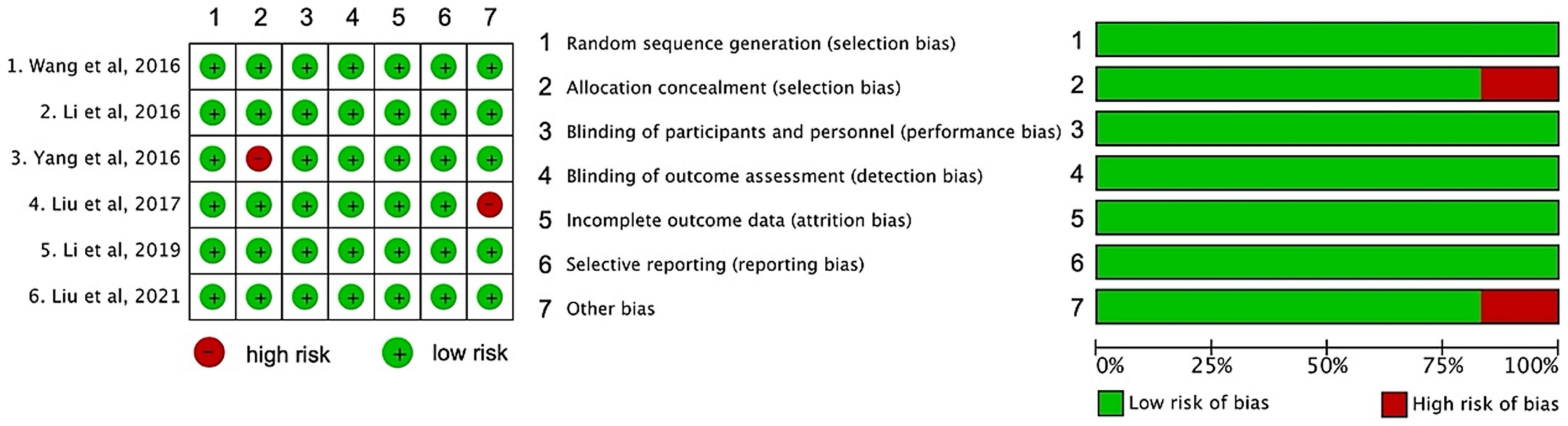
Risk of bias summary of included studies.

Quality assessment was performed using the Cochrane Risk of Bias tool for RCTs and the Newcastle-Ottawa Scale (NOS) for cohort studies. For RCTs (20, 21, 23), we evaluated selection bias, performance bias, detection bias, attrition bias, and reporting bias. For cohort studies (14, 15, 22), NOS assessed selection, comparability, and outcome assessment. This dual approach ensured rigorous evaluation of both study types. As shown in Figure 5, most studies demonstrated a low risk in random sequence generation and incomplete outcome data. However, high-risk issues were identified in allocation concealment and other bias for some studies. Blinding-related biases also showed variations among studies.

## 4 Discussion

DVT remains a significant complication following orthopedic surgery, and this study highlighted the critical role of nursing interventions, including early mobilization and patient education, in reducing the incidence of DVT among postoperative orthopedic patients. Our findings align with recent research, which emphasizes the importance of non-pharmacological strategies in DVT prevention. For instance, the research by Raya-Benítez et al. demonstrated that early mobilization significantly reduces the risk of DVT in hospitalized patients, particularly in the postoperative setting (14). Similarly, Guo et al. found that comprehensive functional exercises combined with patient education effectively lowered the incidence of venous thrombosis after major gynecologic surgery, further supporting the value of nursing-led interventions (15).

The mechanisms by which nursing interventions reduce DVT risk are multifaceted. Early mobilization promotes venous return, reducing stasis in the lower extremities, which is a key factor in thrombus formation. Additionally, patient education enhances awareness of the importance of movement and adherence to preventive measures, thereby addressing modifiable risk factors such as immobility and lack of patient engagement. However, according to the findings from our clinical case, the adherence to the exercise remains a big challenge. Despite the implementation of early mobilizations, the patient researched in our study still developed DVT due to poor compliance, which should be brought by the postoperative pains. This result addresses the importance for considering the individual factors on the patient interventions, such as the aspects on pain management and emotional support, to elevate the adherence and postoperative recovery outcomes.

While postoperative D-dimer is not diagnostic for DVT (21), serial measurements helped characterize the temporal relationship between thrombus formation and fibrin degradation activity (23), providing dynamic insights into coagulation status. The fluctuating D-dimer levels in the patient case unveiled valuable clues into the dynamic nature of thrombosis formation and resolution. Elevated D-dimer levels, as observed in the researched patient, seems to be related with DVT occurrence. This finding keeps consistent with Ramli et al. (7), who identified D-dimer as a reliable biomarker for venous VTE. Continuous D-dimer monitoring may serve as a supplementary tool in high-risk patients with clinical suspicion of DVT, though current guidelines do not recommend routine serial testing. Further studies are needed to validate its role in postoperative care.

The hybrid design reveals a critical insight: while meta-analysis confirms nursing interventions reduce DVT risk at population level, the case illustrates how postoperative pain may compromise guideline adherence in individual patients, leading to DVT despite evidence-based care. This integration of population-level evidence and case-based nuance highlights that while standardized nursing protocols are effective overall, personalized adjustments may be also needed to optimize outcomes in vulnerable patients.

Despite these promising results by timely interventions and D-dimer detection, this study still has several limitations. Firstly, solely one single clinical case limits the generalizability of the findings. Although this case provides valuable insights into the challenges of DVT prevention, larger-scale studies are needed to validate these observations. Secondly, the heterogeneity of the included studies may have influenced the overall results, particularly in terms of intervention protocols and patient populations. Excluding passive mobilization is another limitation, which may benefit immobile patients. Thus active-passive strategies could be incorporated in the studies in the future. In addition, more research should be carried out to standardize intervention measures and include more diverse patient cohorts to enhance the robustness of the findings.

## 5 Conclusion

Nursing interventions such as early mobilization and patient education are found to play a vital role in preventing DVT cases in the orthopedic postoperative recovery process among the patients. This research demonstrates that these interventions significantly reduce the incidence of DVT. However, challenges such as poor patient adherence and variability in intervention delivery must be addressed to maximize their effectiveness. The integration of standardized nursing protocols, tailored to individual patient needs, is essential for optimizing DVT prevention strategies.

Future research should focus on expanding the sample size, standardizing intervention protocols, and exploring innovative approaches to improve patient adherence to the interventions. Additionally, the role of biomarkers such as D-dimer in monitoring DVT risk needs further investigation to be further applied in clinic postoperative practices. By addressing these areas, the quality of postoperative care of the orthopedic patients will be elevated and the burdens of DVT shall be further reduced.

## Data Availability

The raw data supporting the conclusions of this article will be made available by the authors, without undue reservation.
